# Ectopic expression of potato *ARP1* encoding auxin-repressed protein confers salinity stress tolerance in *Arabidopsis thaliana*

**DOI:** 10.1371/journal.pone.0309452

**Published:** 2024-10-17

**Authors:** Sara AlNeyadi, Sajeesh Kappachery, Tanveer Alam Khan, Sameera Karumannil, Mohammed AlHosani, Mayank Anand Gururani

**Affiliations:** Biology Department, College of Science, United Arab Emirates University, Al Ain, UAE; Hainan University, CHINA

## Abstract

Salinity stress is one of the most detrimental factors affecting crop production worldwide. Genetic engineering offers a promising approach for improving agronomic traits and enhancing stress tolerance. In a previous work, several potential candidate genes were identified in potato using large-scale functional yeast screening. In this work, we characterized one of the identified genes, an auxin-repressed protein 1 (*ARP1)*, in transgenic *Arabidopsis* plants. *ARP1* transgenic lines were subjected to salinity stress and compared with wild-type (WT) plants. Compared to WT plants, transgenic *ARP1* lines showed significant improvements in morphological parameters, such as plant height, leaves per plant, root length, and fresh weight. Additionally, biochemical and physiological analyses revealed that the transgenic *ARP1* lines exhibited improved stomatal conductance, reduced electrolyte leakage, increased proline and chlorophyll accumulation, significantly enhanced malondialdehyde accumulation, and antioxidant enzyme activity. Additionally, spectral analysis revealed that transgenic *ARP1* lines had increased photosynthetic capacity compared to WT plants, as indicated by various biochemical parameters and pigment indicators. Transgenic *ARP1* lines also showed improved photosystem (PSII) efficiency compared to WT plants, as demonstrated by detailed chlorophyll fluorescence analyses. Moreover, both *ARP1* lines showed significantly higher expression levels of SOD, CAT, and APX than the WT plants under salt stress. The highest increase in relative expression was observed with SOD (3-fold increase) as compared to their respective WT in both *ARP1* lines. We conclude that potato *ARP1* is a promising candidate gene for the future development of salt-tolerant crops.

## 1. Introduction

One of the most common abiotic stressors is increased soil salinity, which has a negative impact on plant development and growth. Soil salinity affects around one billion hectares (ha) of land in 100 countries, and this figure is expected to rise by 0.3–1.5 million ha annually [1]. Dry and semi-arid regions, such as the United Arab Emirates, were the most affected. Numerous causes contribute to the developing salinity concerns in the region, such as inadequate precipitation, high rates of evaporation, lack of water supplies, and poor management of irrigation [2]. During the 1^st^ phase, soil salinity decreases soil water potential, causing osmotic stress. In previous research [2], excessive salt in the soil prevents plants from absorbing water and other soluble nutrients such as K^+^ and Ca^2+^, which are required for plant development. The second phase, which begins in a few days or weeks depending on the severity of salt stress, occurs when Cl^-^ and Na^+^ ions accumulate (ion toxicity) in various plant tissues. Overaccumulation of Na^+^ and Cl^-^ ions causes nutritional imbalances, metabolic and physiological disorders, membrane disruption, and increased production of reactive oxygen species (ROS) [3]. Collectively, these effects impair essential cellular functions in plants [2]. Salinity stress exacerbates crucial physiological traits in plants, including photosynthesis, stomatal conductance, leaf chlorophyll content, seed germination rate, and other growth-related attributes [4, 5]. Chloroplasts, the organelles in cells that carry out photosynthesis, are particularly vulnerable to harm when exposed to salt stress. Elevated salt concentrations induce thylakoid disarray, damage membranes, make it difficult to distinguish between grana and stroma lamellae, and may even cause chloroplast disintegration [6]. Furthermore, plants under salt stress experience lower water potential, poor photosynthesis, and disruptions in electron transport due to the buildup of sodium (Na^+^) and chloride (Cl^-^) ions within chloroplasts [7]. Like other abiotic stresses, salt-induced stress destabilizes the pigment-protein complex and reduces photosynthetic pigments by increasing the activity of chlorophyllase, producing excessive ROS, or both [8]. Huang et al. [9] found that the expression of *Ndhf* genes linked to light response, *Rbcl* genes linked to dark response, and *Matk* genes connected to chloroplast intron splicing were downregulated in *Eucalyptus robusta* chloroplasts under 150 mM NaCl stress [9]. The intricate relationship between salt stress and water shortage not only impairs plant metabolism but also generates ROS that are harmful to plant systems. Under oxidative stress, studies on Chinese bayberry trees have shown a considerable increase in the activity of the enzymes superoxide dismutase (SOD), ascorbate peroxidase (APX), and catalase (CAT) [10]. Like this, rice seedlings exposed to salt stress exhibit increased levels of H_2_O_2_, MDA, and methylglyoxal (MG) synthesis [11]. Additionally, there was an increase in SOD and Lipoxygenase (LOX) activities and a decrease in CAT activity [12]. In 2018 research, sapodilla rootstocks treated with diluted seawater demonstrated notable increases in APX and CAT activity [13]. Another finding suggests that antioxidant agents are essential to date palm cultivars’ mechanisms for coping with salinity. Salinity-tolerant cultivars show higher levels of enzymatic and non-enzymatic antioxidants accumulating in their leaf and root tissues when exposed to salinity stress [14].

According to Lee et al. [15], the growth-stimulating phytohormone, auxin, controls several processes related to plant growth and development, including vascular differentiation, apical dominance, lateral root formation, shoot elongation, and embryo patterning. The auxin-repressed protein1 (*ARP1*) gene family comprises glycine-rich proteins as well as proteins associated with dormancy. *ARPs*, which are found in higher plants, contribute to plant growth and development by regulating gene expression. Several plant species express *ARP1* in dormant and non-growing tissues [16]. Silencing *GERI/ARP1* increases the vulnerability of plants to infection by the tobacco mosaic virus, *Pectobacterium carotovorum* subsp. carotovora, and *Phytophthora parasitica* var. Nicotianae [16]. The putative *ARP* found in a study on *capsicum annum* was shown to be elevated in response to cold and salt stress, indicating that it may be involved in defensive mechanisms against these environmental stresses [17]. Using a large-scale functional yeast screening approach, Gangadhar et al. [18] identified potential drought tolerance genes in *Solanum tuberosum* (potato) and reported that 20 genes were affected by drought, 14 by salinity; and 11, by heat, drought, or salt stress. In this study, we have cloned into *Arabidopsis* one of these 20 genes, auxin-repressed protein 1 (*ARP1*) into *Arabidopsis*. Although the function of this gene in plant growth, development, and biotic stress resistance have been examined, its relevance in abiotic stress tolerance is unknown. The purpose of this study was to functionally evaluate *ARP1* transgenic Arabidopsis lines under salt stress conditions utilizing thorough physiological, molecular, and biochemical techniques.

## 2. Materials and methods

### 2.1 *In silico* analysis of *ARP1*

A pBLAST search was performed to identify potential orthologs of *ARP1* (NCBI RefSeq gene accession number: JX576266). Subsequently, sequences were aligned using ClustalW to construct a phylogenetic tree. Protein modeling was performed using SwissModel hosted at the Biozentrum, University of Basel (https://swissmodel.expasy.org) and subcellular localization of the protein was predicted using DeepLocPro program, Denmark Technological University (https://services.healthtech.dtu.dk/services/DeepLocPro-1.0/).

### 2.2 Generation of plant material

The full-length coding region of *ARP1* (JX576266), which spans 384 base pairs, was amplified from a potato cDNA library using the appropriate primers (S1 Table). Subsequently, the *ARP1* cDNA was cloned into a plant expression vector, pMDC32, containing the CaMV 35S promoter for transgene expression in plants through LR recombination (LR Clonase II TM enzyme mix, Invitrogen, CA, USA) as described earlier [19].

An *Agrobacterium tumefaciens* mediated floral dip method [20] was employed to generate transformed *A*. *thaliana* (WT) plants overexpressing *ARP1*gene. Putative transgenic plants were selected on half strength Murashige and Skoog medium (PT021, Himedia, India) amended with hygromycin (15 μg mL^-1^) and agar. Presence of the transgene cassette in the genomic DNA was confirmed by PCR amplification of fragments of hygromycin phosphotransferase gene (*hph*) as well as *ARP1* gene using specific primers (S1 Table). Two T3 generation lines of transgenic plants showing highest *ARP1* expression, ARP1-L1 and ARP1-L2, overexpressing the *ARP1* gene, were selected for further experiments. Gene cloning and transgenic plant generation were performed at the Department of Molecular Biotechnology, Konkuk University, Seoul, South Korea.

### 2.3 Salinity stress treatments

Wild-type (WT) and transgenic ARP1-L1 and ARP1-L2 *A*. *thaliana* plants grown under controlled conditions (the plants were grown in a growth chamber set at relative humidity of 50–60%, temperature of 23±2°C and a 16 h/8 h day/night photoperiod) were used for salinity tolerance tests. Initially, seeds of WT and transgenic plants were immersed in sterile distilled water and kept in dark at 4°C for two days for cold treatment. The cold treated seeds were further sown on to seedling trays filled with potting mix (Van Egmond, Naarden, Netherlands) for germination in an illuminated growth chamber (16-h light/8-h dark, temperature 23 ± 1°C). Three-week-old plants were exposed to salt stress by adding 200 mM NaCl solution to the trays underneath the pots containing the plants. From 50 mM NaCl to 200 mM NaCl, each increment of 50 mM was applied for two days to induce salinity stress. Non-stressed, well- ARP1-L1-WW watered Col (WT-WW), or transgenic plants (ARP1-L2-WW) were watered with normal tap water. After two weeks of stress treatment, physiological measurements were taken, and samples were collected to perform gene expression and biochemical analysis to assess the differences in salt tolerance between WT and the transgenic plants. After three weeks of treatment growth parameters were recorded. The collected samples and data were WT-WW (wild-type no salt), WT-NaCl (wild-type NaCl treated), ARP1-L1/ARP1-L2-WW (transgenic no salt), and ARP1-L1/ARP1-L2-NaCl (transgenic salt treated).

### 2.4 Measurement of growth parameters

After three weeks of stress treatment, plant height, number of leaves per plant, fresh weight, and root length of both the control and treated plants were measured. After rinsing the plants with distilled water and drying the roots and shoots on paper towels to eliminate any surface moisture, three repetitions of the experiment were conducted to measure the fresh weight of the plants using an precision balance.

### 2.5 Measurement of stomatal conductance

Stomatal conductance was measured on the adaxial surfaces of fully developed intact rosette leaves using a steady-state diffusion leaf porometer (model SC-1; Decagon Devices, Inc., Pullman, WA, USA). The porometer was calibrated prior to the measurements, and stomatal conductance was assessed under normal laboratory lighting and temperature conditions during the day between 12:00 to 14:00 hours.

### 2.6 Measurement of electrolyte leakage

Electrolyte leakage (EL) was assessed using the method developed by Sullivan and Ross [21]. The leaf discs were placed in a boiling tube containing 10 mL of deionized water, and the initial electrical conductivity (ECa) was measured. The tubes were then subjected to heating at 55°C for 30 minutes in a water bath, after which the electrical conductivity (ECb) was measured again. Then, the tubes were boiled at 100°C for 10 min to obtain the final electrical conductivity (ECc). The percentage of EL was calculated using the formula:

$$\text{Electrolyte}\;\text{leakage}\;\left(\%\right)=\text{ECb}–\text{ECa}/\text{ECc}\;\text{x}\;100.$$

### 2.7 Estimation of chlorophyll a fluorescence

The fully expanded topmost rosette leaves of *A*. *thaliana* plants were dark-adapted for 1h using a clip, following which chlorophyll a (Chl a) fluorescence measurements were taken using a Pocket PEA (Hansatech Instruments Ltd., King’s Lynn, UK). The collected data were then analyzed using the Biolyzer software program based on the "JIP-test equations," as outlined earlier [22]. In summary, parameters such as maximal fluorescence (F_M_) and minimal fluorescence (F_O_) of the sampled leaves were used to calculate the quantum yield of photosystem II (PSII), expressed as the Fv/F_M_ ratio. Furthermore, various photosystem-related parameters, as described in previous studies [23, 24], were derived from the collected data.

### 2.8 Leaf spectrometer measurements

Spectral data obtained from leaves in response to stressors serve as a tool for quantifying plant stress tolerance. Fully grown rosette leaves from each plant were harvested and spectral readings were taken using a CI-710s SpectraVue Leaf Spectrometer (CID Bio-Science, Washington, USA). Subsequently, the recorded transmission, absorption, and reflection data were analyzed to derive various indicators of plant health, stress levels (normalized difference vegetation index (NDVI), plant senescence reflectance index (PSRI), CRI1, water band index (WBI), normalized pigment chlorophyll index (NPCI), ARI1, FRI1, photochemical reflectance index (PRI), chlorophyll content index (CCI), and greenness), and pigment content using the built-in indices provided by the spectrometer.

### 2.9 Estimation of malondialdehyde content

Approximately 500 mg of leaf samples were ground to a powder in liquid nitrogen and then homogenized in 5 mL of 50 mM buffer solution (comprising 0.07% NaH_2_PO_4_·2H_2_O and 1.6% Na_2_HPO_4_·12H_2_O). Following homogenization, the mixture was centrifuged at 20,000 × *g* for 25 minutes at 4°C. Subsequently, 4 mL of 20% trichloroacetic acid containing 0.5% thiobarbituric acid was added to 1 mL of the resulting supernatant. The mixture was then incubated at 95°C for 30 min, cooled on ice, and centrifuged at 10,000 × *g* for 10 min. The absorbance of the supernatant was measured at 532 nm and 600 nm. The absorbance at 600 nm was subtracted from that at 532 nm to correct for nonspecific absorption. The concentration of MDA was then determined using an MDA extinction coefficient of 155 mM^–1^cm^–1^, as described earlier [25].

### 2.10 Determination chlorophyll content

To determine the chlorophyll content, the leaf samples collected were ground in 2 mL of 80% acetone and kept at 4°C overnight in the dark. Following dark incubation, the mixture was centrifugated at 3,000 × *g* for 5 min, the absorbance of the supernatant was recorded at 663, 645, and 652 nm, and chlorophyll content was determined as described previously by Arnon [26].

### 2.11 Proline estimation

The proline content of the leaves was estimated using a previously outlined colorimetric method [22]. Initially, approximately 500 mg of leaf sample was ground into a fine powder in liquid nitrogen, followed by homogenization in 10 mL of 3% aqueous sulfosalicylic acid. Subsequently, equal volumes (2 mL each) of the filtered homogenate, acid ninhydrin, and glacial acetic acid were combined and incubated at room temperature. After 1h, the reaction was stopped by cooling the tubes on ice. The chromophore-containing phase was extracted with 4 mL of toluene, and the absorbance was measured at 520 nm. Finally, proline concentration in the samples was determined by plotting the absorbance values against a standard curve constructed using known concentrations of proline.

### 2.12 Estimation of ROS enzyme activity

Leaf samples were crushed in a chilled mortar and pestle in the presence of liquid N_2_. Total protein was extracted with a buffer containing 0.2 M potassium phosphate buffer (pH 7.5), 50% (v/v) glycerol, 16 mM MgSO_4_, 0.2 mM phenyl methyl sulfonyl fluoride, and 0.2% polyvinylpolypyrrolidone and centrifuged at 13,000 × *g* for 30 min at 4°C. The supernatant was collected, and the protein content was determined as previously described (Bradford, 1976). Specific enzyme activities for ascorbate peroxidase (APX), catalase (CAT), superoxide dismutase (SOD), and glutathione reductase were determined as previously described [22, 27].

### 2.13 Quantitative PCR analysis of genes related to ROS scavenging, photosynthesis, and salinity response

Total RNA was isolated from different samples using an ISOLATE II RNA Plant Kit (Bioline BIO-52077). Five hundred nano gram of total RNA per sample was used for cDNA synthesis using SensiFAST cDNA Synthesis Kit (Bioline, BIO-65053). The cDNA was diluted to 1:4 using nuclease-free water. Five microliters of diluted cDNA were used per reaction (total volume 20 μl) as a template for qRT-PCR. The qRT-PCR reaction was run on QuantStudio^™^ 5 Real-Time PCR System (A34322, Thermo Fisher) using SensiFAST SYBR Lo-ROX Kit (BIO-94005, Bioline, UK) master mix. Glyceraldehyde-3-phosphate dehydrogenase gene (AT1G13440) was used as the housekeeping gene and expression levels of different genes related to ROS scavenging were analyzed. Relative transcript level of genes was calculated using the 2^–ΔΔCt^ method [28]. The mean values of relative gene expression were calculated based on data collected from four biological replicates. The specific primer sets used for the qRT-PCR are listed in S1 Table.

### 2.14 Statistical analyses

All experiments were conducted using a completely randomized block design. The experiments were repeated at least three times with 15–20 replicates. Statistical analyses were performed using Origin 8.0 software program.

## 3. Results

### 3.1. *In silico* analyses of *ARP1* gene

**Fig 1:**
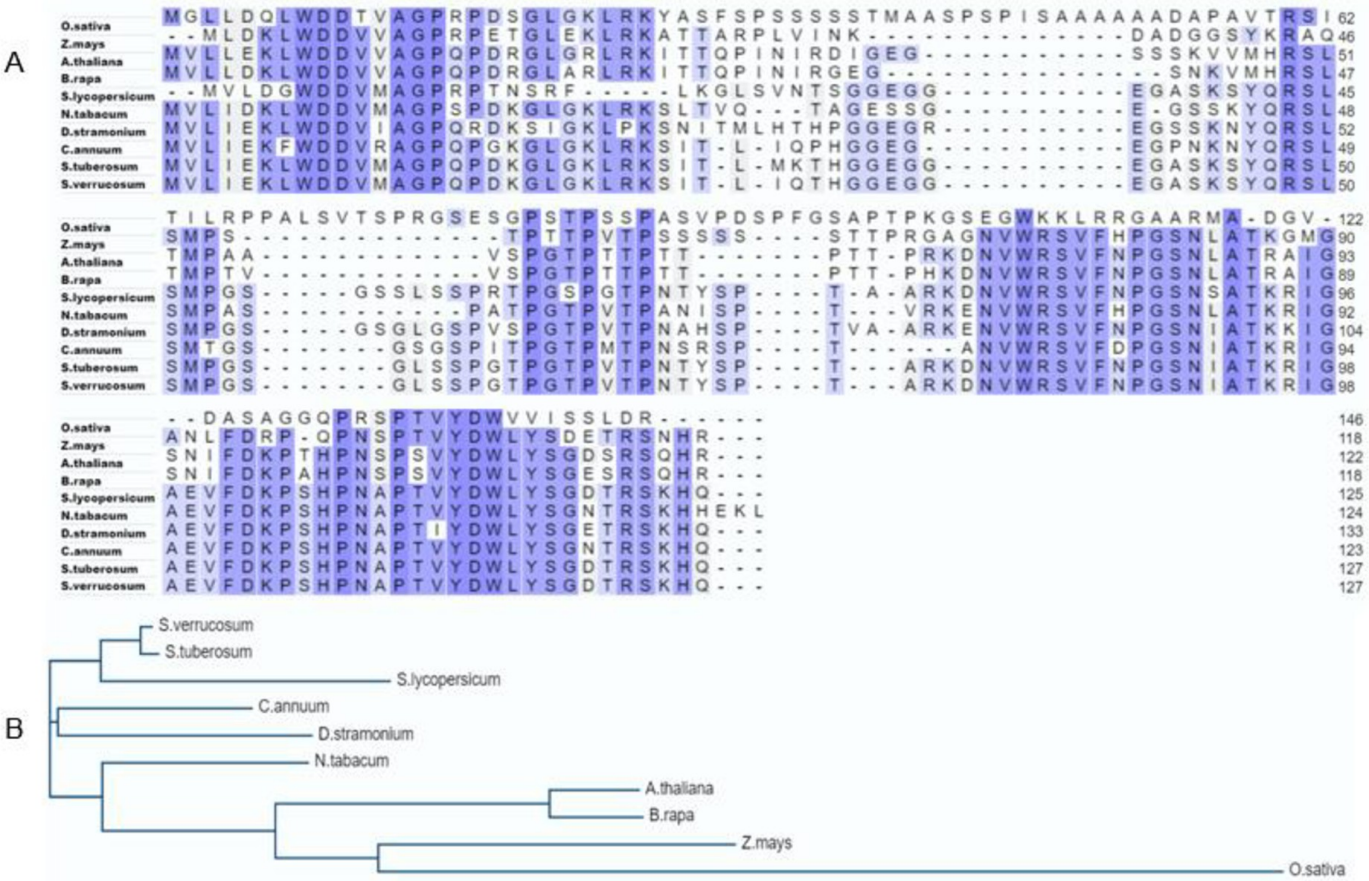
**A.** Multiple sequence alignment and **B.** phylogenetic analysis of auxin-repressed protein 1 (*ARP1*) from *S*. *tuberosum* (AFW90622.1) and other plant species [*S*. *verrucosum* (XP_049361215.1), *S*. *lycopersicon* (XP_019068303.1), *D*. *stramonium* (MCD9642534.1), *C*. *annuum* (XP_016570509.1), *N*. *tabacum* (AAS76635.1), *A*. *thaliana* (NP_564305.1), *B*. *rapa* (XP_009113675.2), *O*. *sativa* (XP_015612469.1) and *Z*. *mays* (NP_001152232.2)].

**Fig 2:**
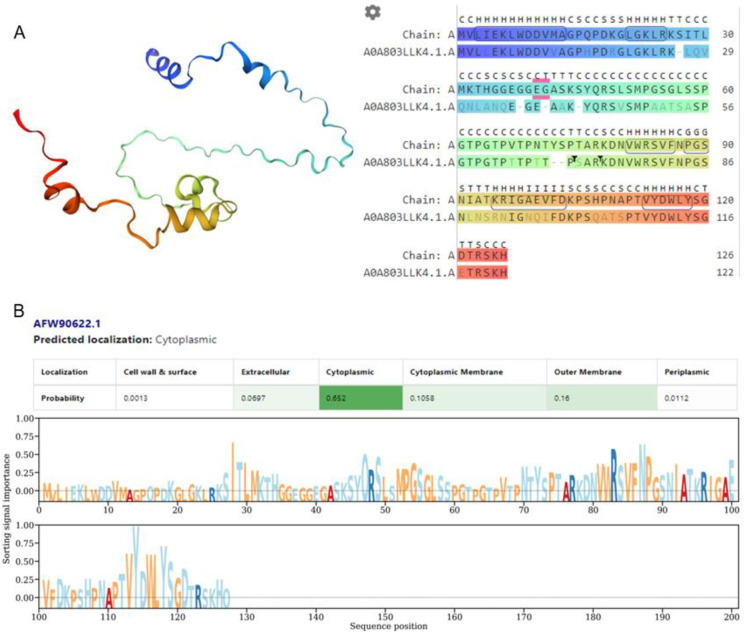
**A.** Protein modeling of potato auxin-repressed protein 1 (*ARP1*) performed using SwissModel, Biozentrum, University of Basel (https://swissmodel.expasy.org). **B.** Subcellular localization of auxin-repressed protein 1 (*ARP1*; NCBI ID: AFW90622.1) was predicted using DeepLocPro program, Denmark Technological University (https://services.healthtech.dtu.dk/services/DeepLocPro-1.0/).

Multiple sequence alignment results revealed that *ARP1* is a conserved protein with strong sequence similarity to dicots from the Brassicaceae and Solanaceae families, as well as monocot grasses like rice and maize (Fig 1). Protein modeling showed a sequence identity of 70% with A0A803LLK4.1. A (ARP AlphaFold DB model of A0A803LLK4_CHEQI (gene: A0A803LLK4_CHEQI; organism: *Chenopodium quinoa* (quinoa)) (Fig 2A). Further an *in silico* subcellular localization investigation demonstrated that the protein coded by the *ARP1* gene will be primarily expressed in the cytoplasm (Fig 2B).

### 3.2. Molecular study of *Arabidopsis* plants that express the potato *ARP1* gene

**Fig 3:**
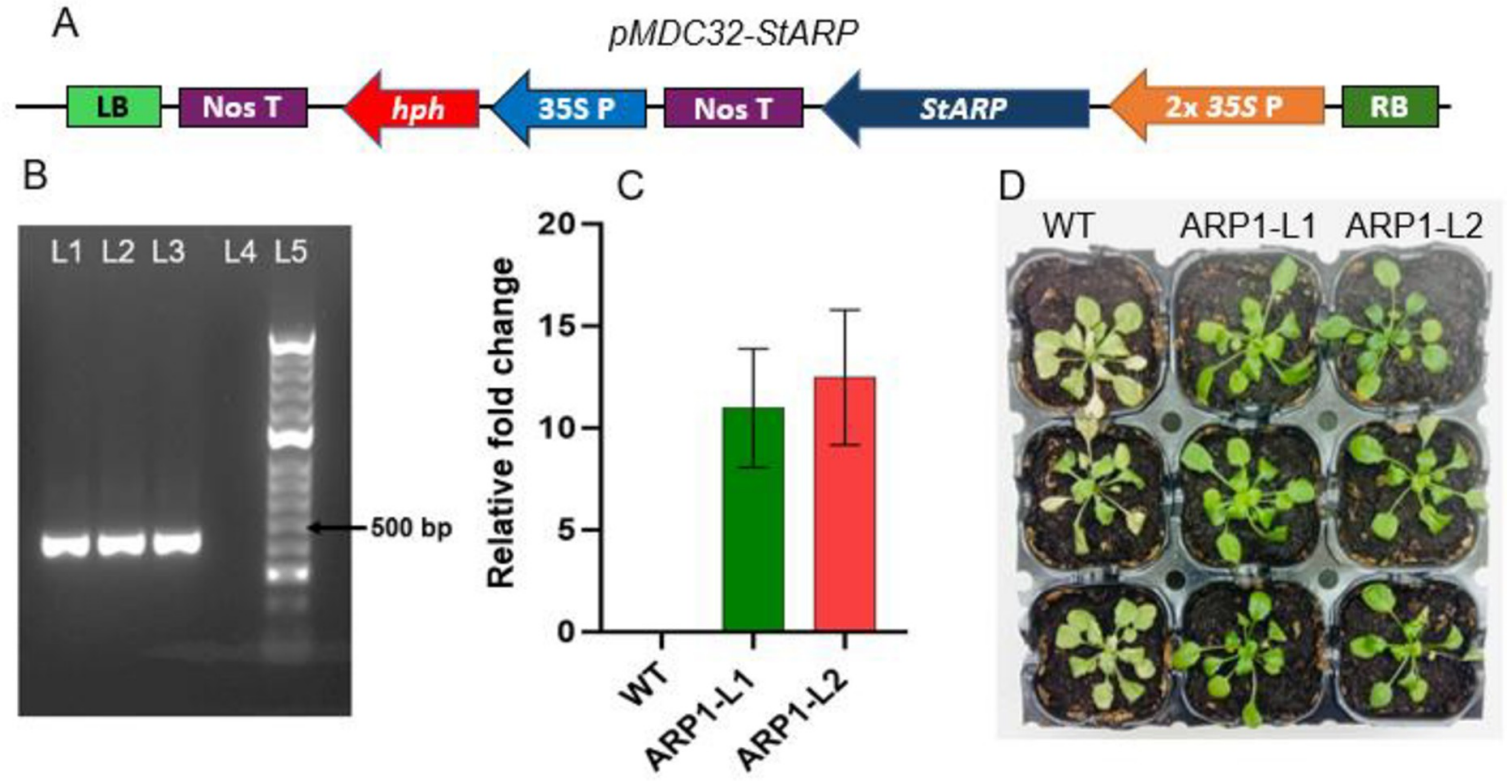
**A.** T-DNA map of pMDC32-*StARP* construct used to generate *ARP1* transgenic lines. **B.** Morphological differences between *Arabidopsis* wild-type (WT) and transgenic *ARP1* lines (T3 generation) after 10 days of exposure to 200 mM NaCl stress. **C.** PCR amplification of hygromycin phosphotransferase gene (412 bp) from genomic DNA of two hygromycin-resistant transgenics lines. L1 and L2 corresponds to ARP1-L1 and ARP1-L2, L3 and L4 corresponds to positive (DNA from plants harboring vector pMDC32 only) and negative control (WT) plants and L5 is DNA ladder. **D.** Relative expression of *StARP* gene in wild-type (WT) and transgenic *ARP1* lines. The expression of the *ARP1* was normalized to the *GAPDH*, with WT plants used as the calibrator (n = 5).

Transgenic *A*. *thaliana* plants expressing the potato *ARP1* gene were generated using a pMDC32 vector construct having *StARP1* and the hygromycin resistance marker gene *hph* (Fig 3A). Two of the T1 generation plants resistant to hygromycin (selected on ½ MS media plates containing hygromycin) were confirmed to have *St-ARP1* gene by PCR amplification of a 136 bp long *ARP1* gene fragment (S1 Raw images) from their genomic DNA (gDNA) extract, using ARP1_F and ARP1_R primers (S1 Table). Further two of the T3 lines (100% resistant to hygromycin), ARP1-L1 and ARP1-L2 separately generated from these T1 plants were selected for further characterization (S1 Raw images). The *hph* gene-specific primers showed no amplification in WT but were amplified in WT *A*. *thaliana* plants but were amplified in both the selected transgenic lines (ARP1-L1 and ARP1-L2), validating the presence of *hph* marker gene in both transgenic lines (Fig 3B). In addition, RT-PCR analysis confirmed the expression of the *StARP-1* gene in both the lines, whereas no expression was noted in WT plants (Fig 3C). Phenotypic differences were visible between the WT and *ARP1* transgenic lines after two weeks of salinity stress induced by 200 mM NaCl (Fig 3D). Although the WT plants turned from pale green to yellow, the *ARP1* transgenic plants appeared greener and healthier.

### 3.3. Evaluation of transgenic *ARP1* lines under salinity stress

**Fig 4:**
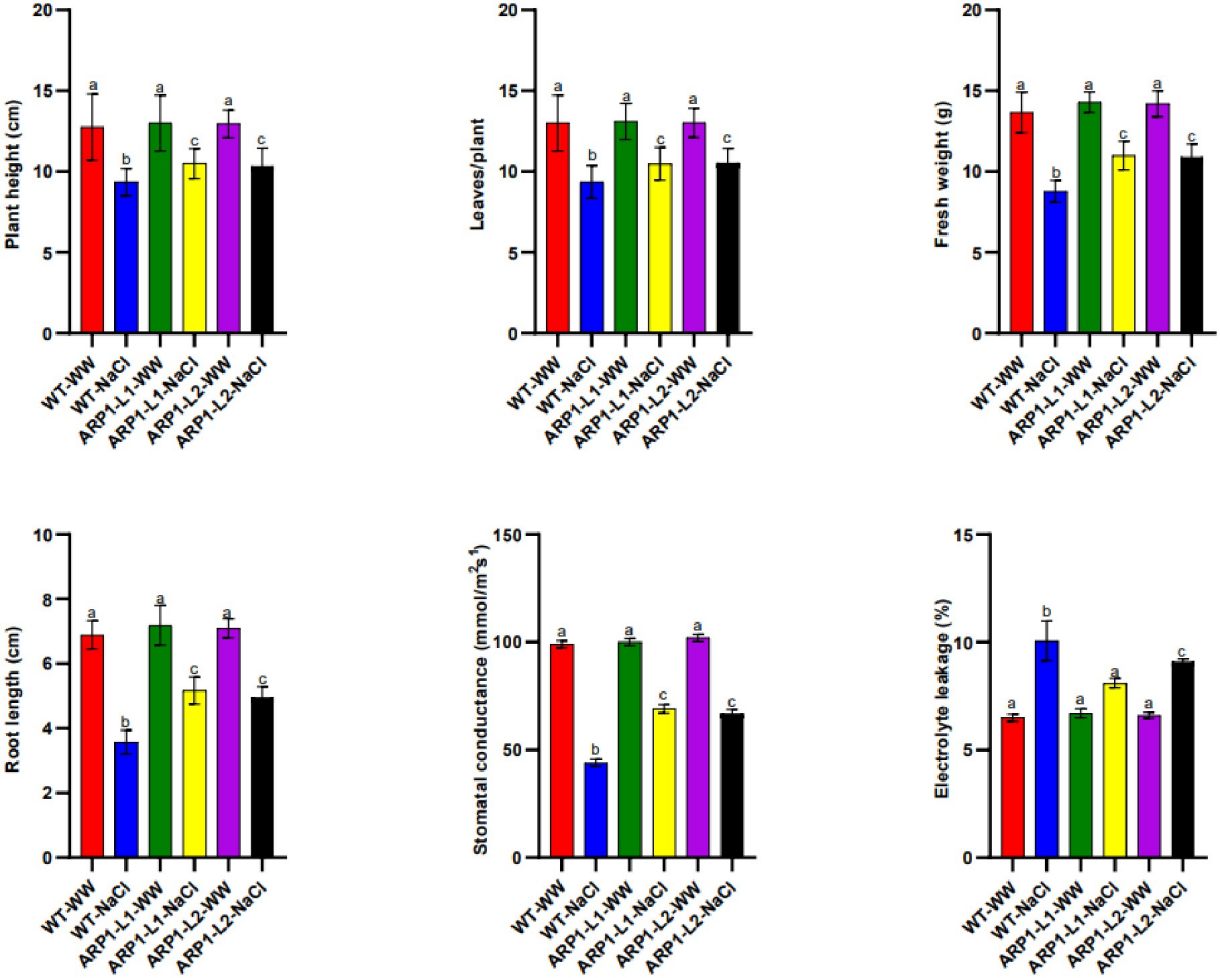
Assessment of (A) plant height, (B) leaves/plant, (C) fresh weight, (D) root length, (E) stomatal conductance, (F) electrolyte leakage in wild-type (WT) and transgenic *ARP1* lines under non-stress (WT-WW, ARP1-L1-WW and ARP1-L2-WW) and salinity (WT-NaCl, ARP1-L1-NaCl and ARP1-L2-NaCl) conditions. Different letters above bars indicate significant differences (p≤0.05) between plant types under non-stress and stress conditions using a Tukey’s honestly significant difference test (n = 5). Bars represent the means ± standard deviation.

WT-WW and ARP1-L1 and ARP1-L2 transgenic lines exposed to salinity stress (200 mM) showed a significant increase in stress tolerance in ARP1-L1 and ARP1-L2 transgenic lines when compared with WT. Salinity stress led to a decrease in plant height in WT-NaCl and transgenic ARP1-L1-NaCl and ARP1-L2-NaCl by 26.66%, 19.23%, and 20% respectively (Fig 4A); leaves/plant in WT-NaCl and transgenic ARP1-L1-NaCl and ARP1-L2-NaCl by 28%, 20%, and 19% respectively (Fig 4B); fresh weight in WT-NaCl and transgenic ARP1-L1-NaCl and ARP1-L2-NaCl by 35.67%, 23.09%, and 23.18% respectively (Fig 4C); and root lengths in WT-NaCl and transgenic ARP1-L1-NaCl and ARP1-L2-NaCl by 48.04%, 28.09%, and 30% respectively (Fig 4D) compared with the respective values in WT-WW and ARP1-L1-WW and ARP1-L2-WW plants. Salinity stress treatment reduced stomatal conductance by 55.5% in WT, 31% in ARP1-L1, and 34.3% in ARP1-L2 plants (Fig 4E), whereas EL increased 54.7% in WT, 20.9% in ARP1-L1, and 37.8% in ARP1-L2 (Fig 4F). The ARP1-L1 and ARP1-L2 transgenic lines were substantially greener and healthier than the WT plants (Fig 3D), demonstrating that *ARP1* plays an advantageous role in reducing salt toxicity in transgenic *Arabidopsis* plants. This difference was evident between the control and both of transgenic *ARP1* lines exposed to salinity.

### 3.4. Leaf spectral analysis in WT and *ARP1* transgenic lines under salt stress

**Fig 5:**
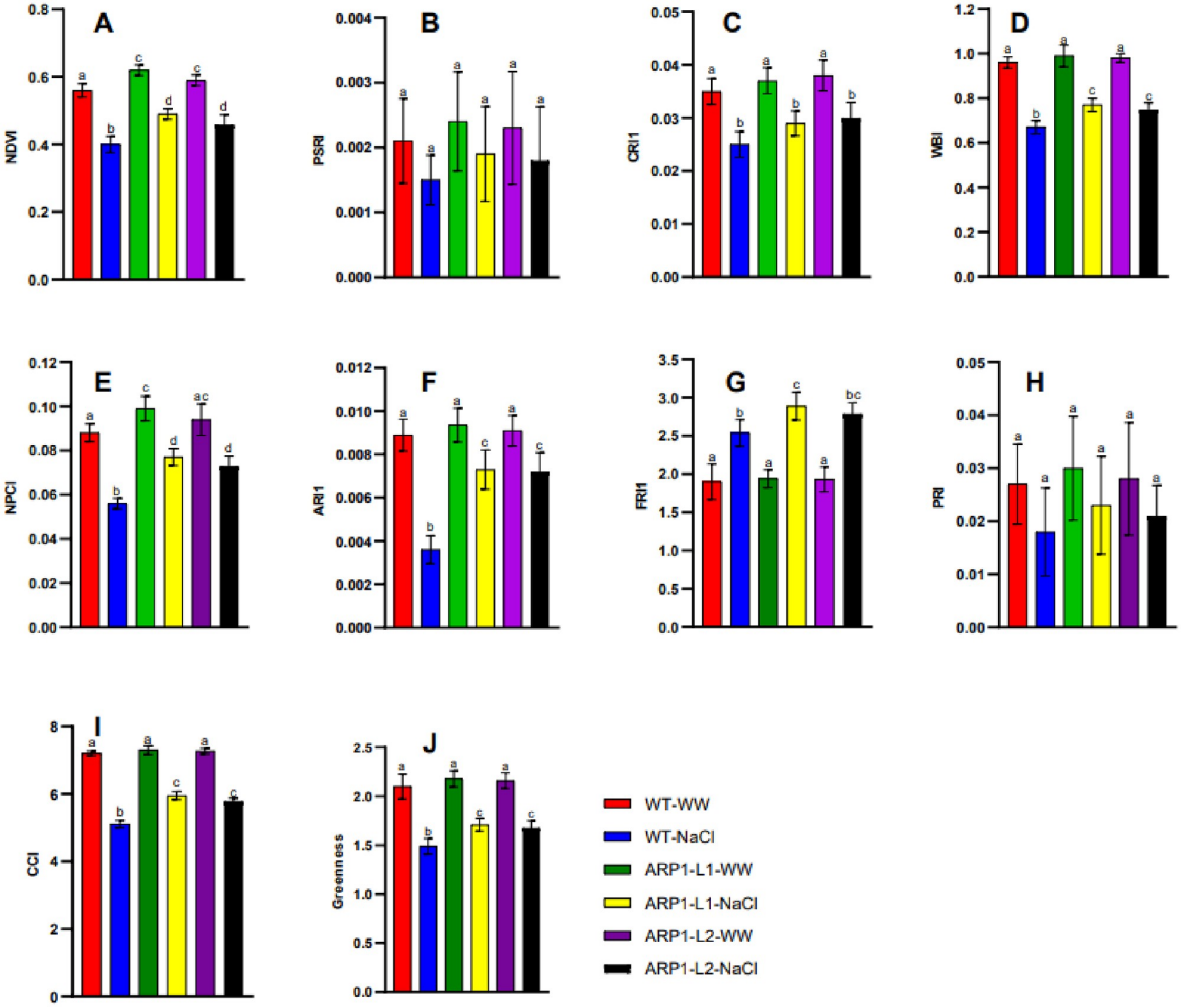
Assessment of (A) NDVI, (B) PSRI, (C) CRI1, (D) WBI, (E) NPCI, (F) ARI1, (G) FRI1, (H) PRI, (I) CCI, and (J) Greenness in wild-type (WT) and transgenic *ARP1* lines under non-stress (WT-WW, ARP1-L1-WW and ARP1-L2-WW) and salinity (WT-NaCl, ARP1-L1-NaCl and ARP1-L2-NaCl) conditions. Different letters above bars indicate significant differences (p≤0.05) between plant types under non-stress and stress conditions using a Tukey’s honestly significant difference test (n = 5). Bars represent the means ± standard deviation.

Plant stress is reflected to spectral vegetation indices. These parameters can be divided into three categories: water status, greenness, and xanthophyll index. Leaf spectral indices, including the NDVI, PSRI, carotenoid reflectance index 1 (CRI1), WBI, NPCI, anthocyanin reflectance index 1 (ARI1), flavanols reflectance index 1 (FRI1), PRI, CCI, and greenness were used to further illustrate the physiological states of the WT and *ARP1* transgenic lines. However, reduced the NDVI content in WT-NaCl, transgenic ARP1-L1-NaCl and ARP1-L2-NaCl by 27.1%, 19.5%, and 20.6%, respectively; NPCI by 35.8%, 21.6%, and 21.9%, respectively; and PRI values by 30%, 22%, and 22.4%, respectively, in relation to the corresponding values in ARP1-L1-WW, ARP1-WW, and ARP1-L2-WW plants (Fig 5A–5J). Moreover, salt stress also decreased PSRI in WT-NaCl and transgenic ARP1-L1-NaCl and ARP1-L2-NaCl by 26.2%, 18.3%, and 19.1%, respectively; ARI 1 by 28.5%, 21.6%, and 20.4%, respectively; CCI values by 29.2%, 21%, and 20.1%, respectively; and WBI values by 30.1%, 22.1%, and 22.5%, correspondingly related with the respective values in WT-WW, ARP1-L1-WW, and ARP1-L2-WW plants. These findings indicated that when subjected to salt stress, both transgenic *ARP1* lines accumulated more photosynthetic pigments and showed increased photosynthetic activity.

### 3.5. Evaluation of photosynthetic pigment and PSII efficacy in salinized WT and *ARP1* transgenic lines

**Fig 6:**
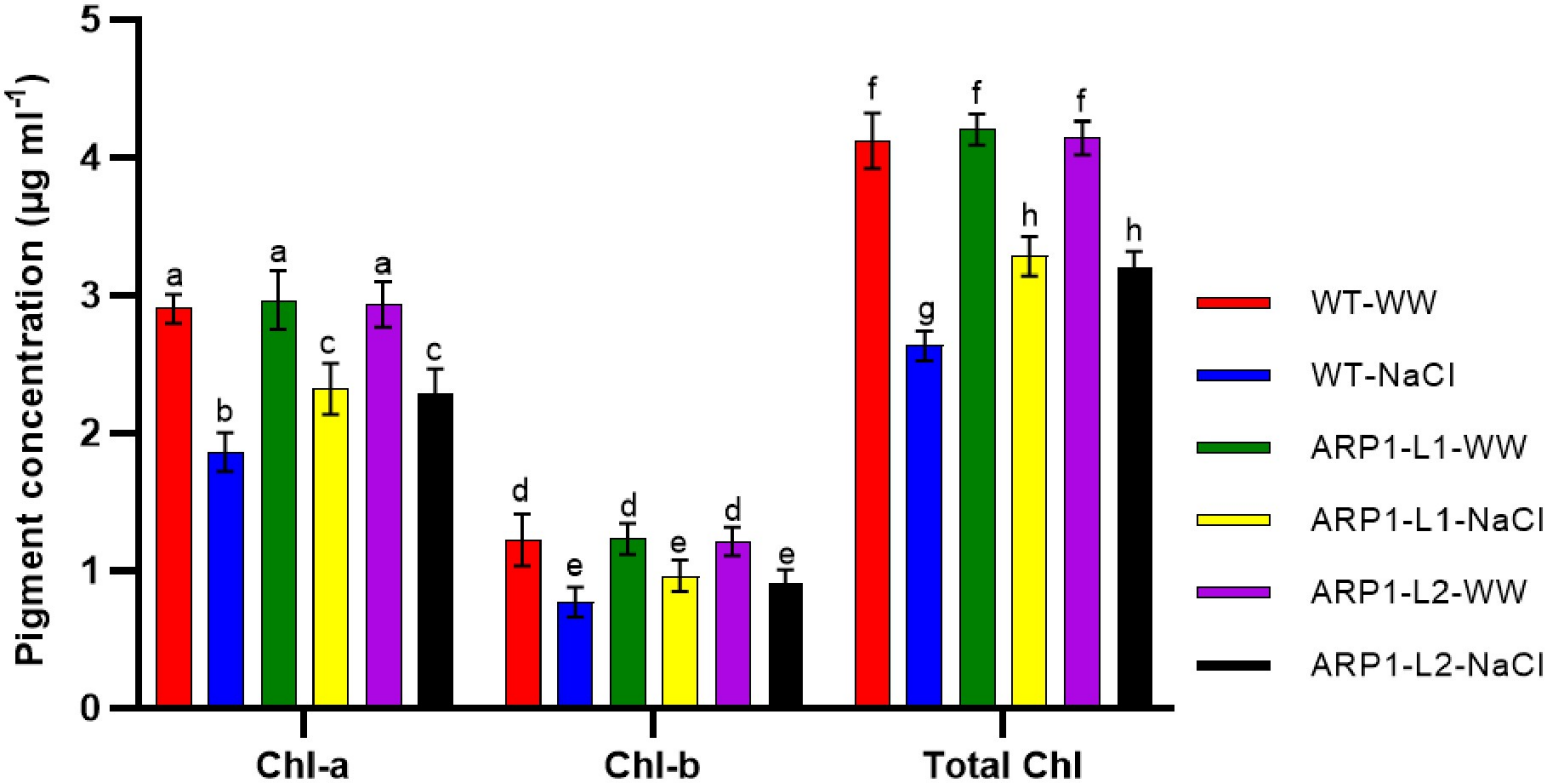
Assessment of Chl a, Chl b, and total chlorophyll in wild-type (WT) and transgenic *ARP1* lines under non-stress (WT-WW, ARP1-L1-WW and ARP1-L2-WW) and salinity (WT-NaCl, ARP1-L1-NaCl and ARP1-L2-NaCl) conditions. Different letters above bars indicate significant differences (p≤0.05) between plant types under non-stress and stress conditions using a Tukey’s honestly significant difference test (n = 5). Bars represent the means ± standard deviation.

The assessment of chlorophyll in the transgenic lines of WT and *ARP1* revealed additional physiological variations between these plants. Following salt-induced stress, a discernible difference was observed between the chlorophyll contents of the WT and *ARP1* lines (Fig 6). In WT-NaCl, transgenic ARP1-L1-NaCl, and ARP1-L2-NaCl, salinity reduced the content of Chl a by 35.86%, 21.62%, and 21.84%, respectively; Chl b by 36.88%, 21.95%, and 24.79%, respectively; and total Chl content by 36.16%, 21.90%, and 22.70%, respectively, in comparison to the corresponding values in WT-WW, ARP1-L1-WW, and ARP1-L2-WW plants (Fig 6). Salinity negatively impacting the photosynthetic pigments of both WT plants and transgenic *ARP1* lines plants, the quantities of chl a, chl b, and total chl in the leaves of ARP1-NaCl plants were higher than those in the leaves of WT-NaCl plants. These results suggest that under salinity-induced oxidative stress, transgenic *ARP1* lines accumulate more photosynthetic pigments, and so have increased photosynthetic activity.

**Fig 7:**
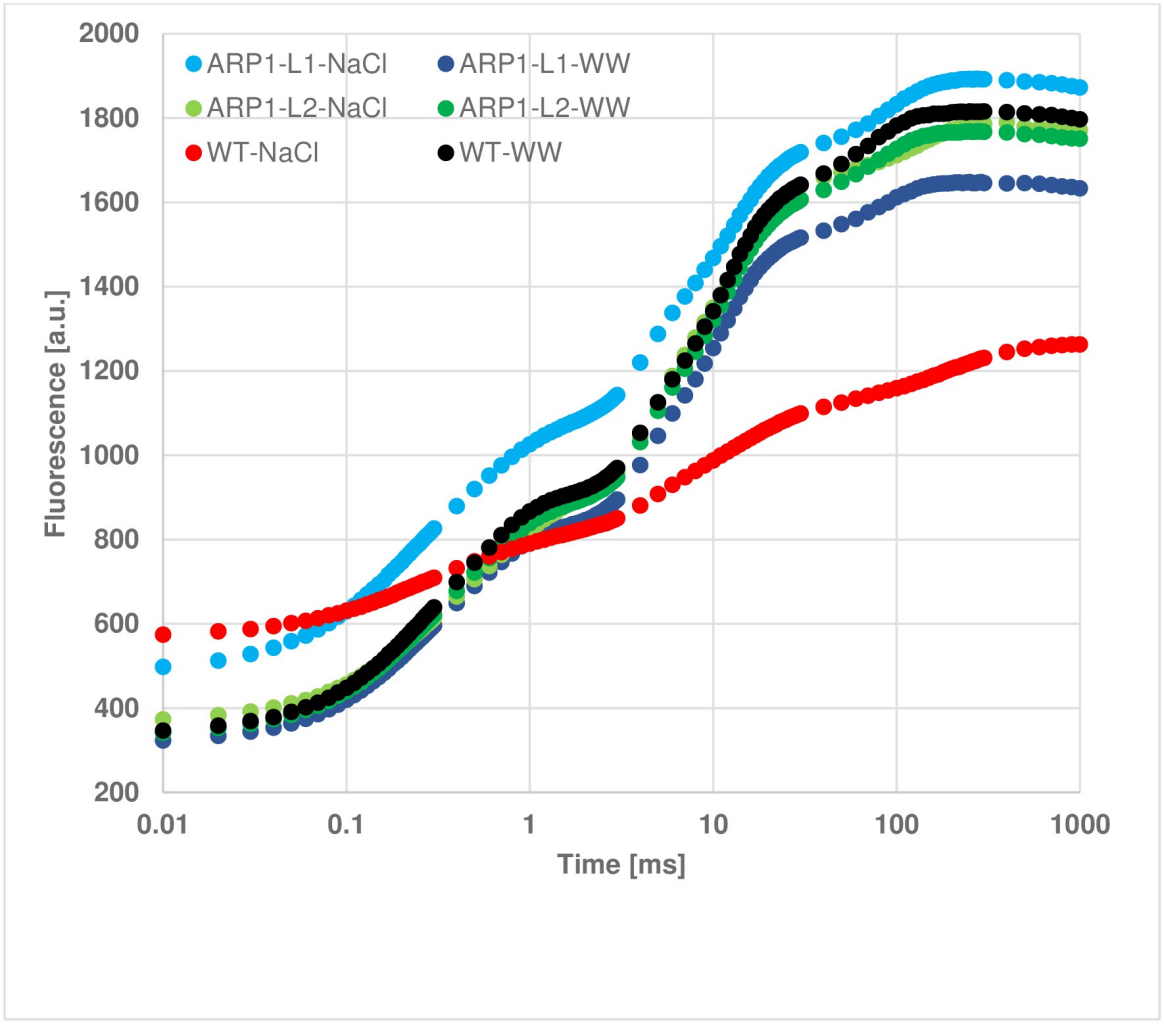
Fast chlorophyll A fluorescence kinetics (OJIP) in dark-adapted *Arabidopsis* leaves from the six experimental groups. The transient polyphasic curves for each line represent the average of 21 measurements, obtained from three replicates, each containing seven plants with respective groups.

**Fig 8:**
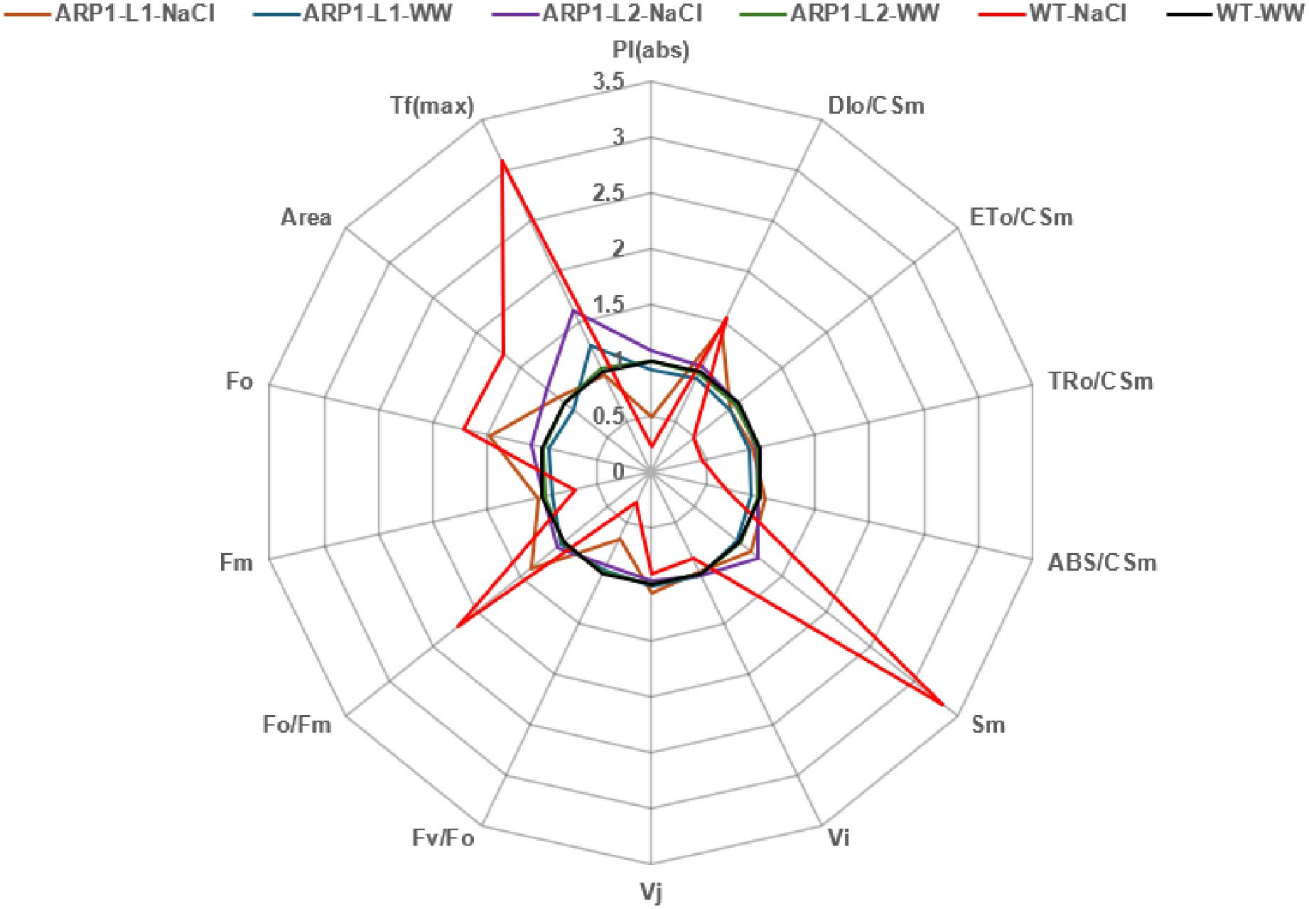
Radar plot showing various biophysical parameters of PSII. The details of the parameters studied are shown in S2 Table. The values of each parameter for each line represent the average of 21 measurements, obtained from three replicates, each containing seven plants with respective groups.

**Fig 9:**
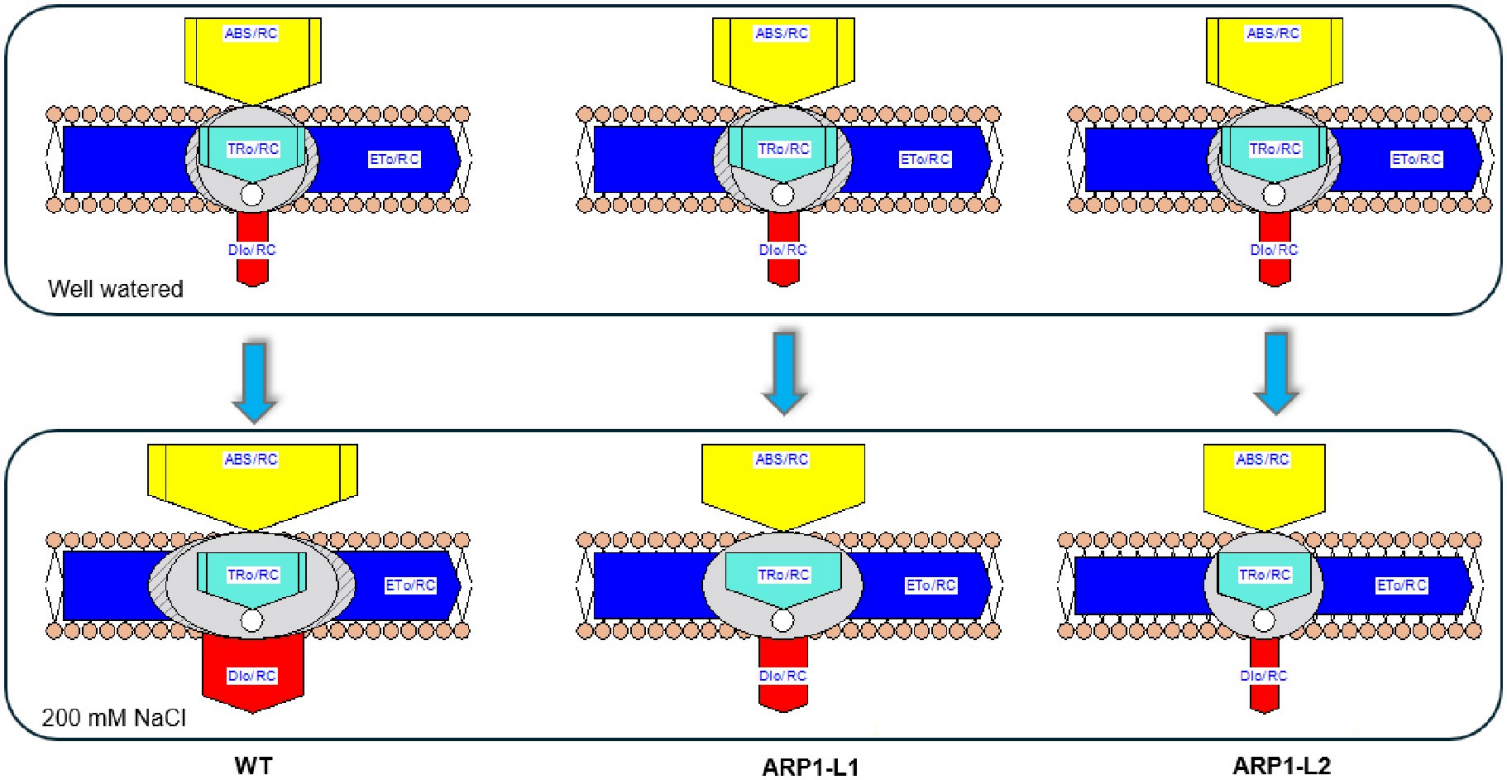
Energy pipeline leaf model of phenomenological fluxes (per reaction center; RC) in six groups. Relative changes in arrow width depict the value of each parameter. The values of each parameter for each line represent the average of 21 measurements, obtained from three replicates, each containing seven plants with respective groups.

Next, we used the JIP test parameters (Table 1; Figs 7–9), which are complex markers of PSII efficiency under stress and plant photosynthetic ability, to quantify the kinetics of Chl a fluorescence. Fig 7 shows the typical fluorescence transient curves of the WT and *ARP1* lines under normal and stress conditions. The fluorescence curves indicated that the plants were photosynthetically active. A declining curve in the WT-NaCl plant indicated that these WT plants suffered the most severe damage to PSII components under salinity stress. The radar plot (Fig 8) further revealed that damage to PSII components was most severe in the WT-NaCl group, as indicated by the various PSII biophysical parameters studied. Phenomenological energy flux diagrams, including ABS/reaction centers (RC), TRo/RC, ETo/RC, and DIo/RC, are shown in Fig 9. These fluxes provide insight into the response of active RCs to light. In particular, ABS/RC indicates an increase in the number of active RCs, TRo/RC indicates the trapped electron flux per reaction center, and ETo/RC represents the electron transport flux per reaction center. Additionally, DIo/RC reflects the total energy dissipated per reaction center. The magnitude of each parameter is represented by varying widths of the arrows. These parameters clearly demonstrated that ARP1-L1 suffered significantly less damage under salinity stress than the WT plants. Based on these analyses, we observed that overexpression of the *ARP1* gene under salinity stress improved both the photochemistry of photosystem II and the performance indices. Salinity reduced the energy absorption-based performance index (PI_ABS_) by 79%, the maximum quantum yield for primary photochemistry (φPo) by 33.69%, and the quantum yield for electron transport (φEo) by 30.76% when comparing WT-NaCl plants to WT-WW plants. Furthermore, we found that under non-stress and saline conditions, *ARP1* lines expression in *A*. *thaliana* sustained the Fv/Fm of PSII; however, the Fv/Fm ratio in WT-NaCl plants decreased by 70.83% in comparison to WT-WW plants (Table 1). In contrast, transgenic ARP1-L1-NaCl plants showed no discernible increase in comparison to ARP1-L1-WW plants, although the specific energy flux, comprising the total energy dissipated per reaction center (DIo/RC), showed a 3.5-fold increase in WT-NaCl plants compared to WT-WW plants. These findings were consistent with the stomatal apparatus performance values. Transgenic ARP1-L1-NaCl plants exhibited 56.81% greater stomatal conductance than WT-NaCl plants (Fig 4E). Altogether, the stomatal conductance values and kinetics of chlorophyll a fluorescence suggest that salinity has a deleterious effect on PSII, which in turn prevents electron transport at the PSII donor site in WT-NaCl plants. In contrast, ARP1-L1-NaCl transgenic plants showed increased photosynthetic capacity and improved PSII efficiency by inhibiting salinity-induced PSII damage.

**Table 1:** Effect of salinity on chlorophyll a florescence kinetics in wild-type (WT) and transgenic *ARP1* lines in *Arabidopsis thaliana* plants.

	WT-WW	WT-NaCl	ARP1-L1-WW	ARP1-L1-NaCl	ARP1-L2-WW	ARP1-L2-NaCl
**PI(abs)**	1.0864	0.2274	0.9204	0.4883	0.9989	1.0835
**DIo/RC**	0.9454	3.3899	1.0578	1.632	0.9965	0.95
**ETo/RC**	0.983	1.0752	1.0172	1.0205	0.9849	0.8883
**TRo/RC**	0.9758	1.0214	1.0247	1.0685	0.9859	0.8731
**ABS/RC**	0.9691	1.5314	1.0319	1.1898	0.9882	0.8896
**PHI(Do)**	0.9755	2.2137	1.0251	1.3716	1.0084	1.0678
**PHI(Eo)**	1.0144	0.702	0.9858	0.8577	0.9968	0.9984
**PSIo**	1.0074	1.0526	0.9927	0.955	0.999	1.0175
**PHI(Po)**	1.0069	0.667	0.9931	0.8981	0.9977	0.9814
**Fv/Fo**	1.0324	0.3013	0.9686	0.6548	0.9897	0.9193
**Fo/Fm**	0.9755	2.2137	1.0251	1.3716	1.0084	1.0678

### 3.6. Evaluation of biochemical parameters

**Fig 10:**
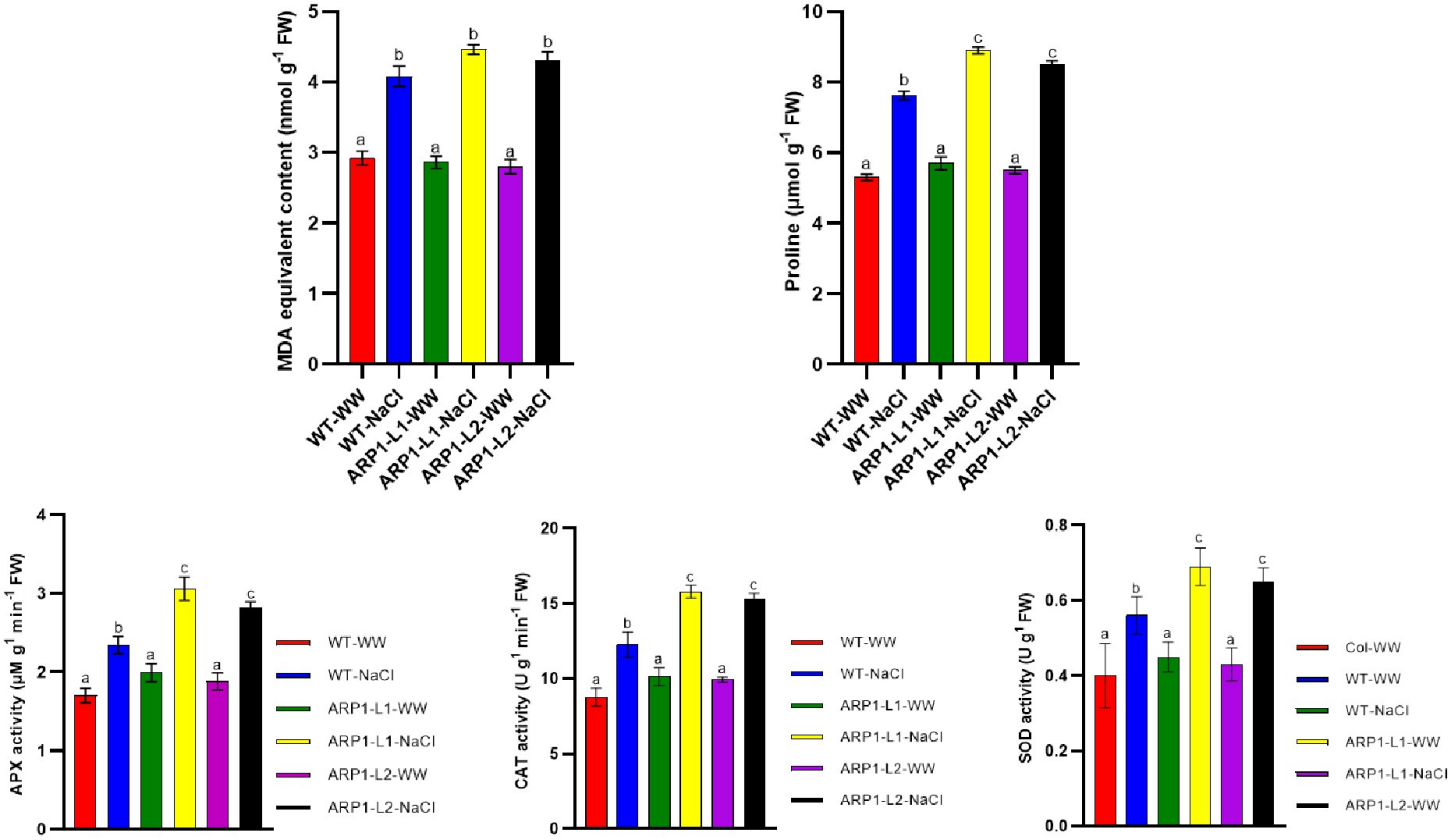
Assessment of (A) malondialdehyde content, (B) proline, (C) APX activity, (D) CAT activity and (E) SOD activity in wild-type (WT) and transgenic *ARP1* lines under non-stress (WT-WW, ARP1-L1-WW and ARP1-L2-WW) and salinity (WT-NaCl, ARP1-L1-NaCl and ARP1-L2-NaCl) conditions. Different letters above bars indicate significant differences (p≤0.05) between plant types under non-stress and stress conditions using a Tukey’s honestly significant difference test (n = 5). Bars represent the means ± standard deviation.

The MDA content, a measure of lipid peroxidation, in WT-NaCl plants by 39.72% compared to WT-WW plants, and in ARP1-L1-NaCl plants by 55.94% compared to ARP1-L1-WW plants (Fig 10A). Plant adaptation to stress is positively indicated by proline content. Proline accumulation increased in response to salt stress compared to that in the control plants (Fig 10B). We observed that salinity induced 43.30%, 56.14%, and 54.54% increases in proline levels in WT-NaCl, ARP1-L1-NaCl, and ARP1-L2-NaCl transgenic lines, compared with the respective values in WT-WW, ARP1-L1-WW, and ARP1-L2-WW plants (Fig 10B). These findings suggest that transgenic plants under saline conditions exhibited superior membrane integrity and osmotic adjustment compared to WT plants because of the ectopic expression of the *ARP1* gene in *A*. *thaliana*.

### 3.7. Analysis of antioxidant enzyme activities and gene expression in transgenic lines of *ARP1*

**Fig 11:**
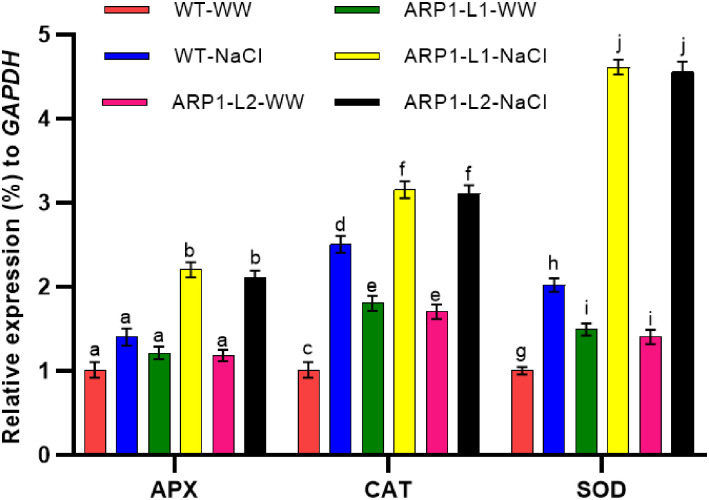
Expression of three antioxidant enzyme-encoding genes in wild-type (WT) and transgenic *ARP1* lines under non-stress (WT-WW, ARP1-L1-WW and ARP1-L2-WW) and salinity (WT-NaCl, ARP1-L1-NaCl and ARP1-L2-NaCl) conditions. Different letters above bars indicate significant differences (p≤0.05) between plant types under non-stress and stress conditions using a Tukey’s honestly significant difference test (n = 5). Bars represent the means ± standard deviation.

In plants exposed to salt, ARP1-L1-NaCl plants showed greater activity of antioxidant enzymes, such as catalase (CAT), ascorbate peroxidase (APX), and superoxide dismutase (SOD) (Fig 10C–10E). APX, SOD, and CAT are major enzymes in the ROS-scavenging system. Transgenic ARP1-L1 and ARP1-L2 lines treated with salt stress had significantly higher APX activity than the WT plants (Fig 10C). In ARP1-L1-NaCl plants, compared with ARP1-L1-WW plants, the enzyme activities of the APX and CAT were 81.81% and 75.1% higher, respectively (Fig 10C and 10D). These enzyme activity analyses were further confirmed by expression analysis of the genes encoding these antioxidant enzymes. When exposed to salt stress, both *ARP1* lines displayed noticeably higher expression levels of SOD, CAT, and APX than the WT plants (Fig 11). The highest increase in relative expression was observed with SOD (3-fold increase) as compared to their respective WT in both *ARP1* lines. However, for CAT and APX, the increase was more than 2-fold in the WT in both *ARP1* lines. This suggests that ectopic expression of *ARP1* confers a greater antioxidant capacity and provides additional evidence of *ARP’s* ability to enhance the ROS-scavenging capacity in transgenic *ARP1* plants exposed to salinity.

## 4. Discussion

Plant cultivation faces a major difficulty due to soil salinization, which is caused by inappropriate agricultural practices and global climate change [27]. Salinization hampers the movement of essential nutrients and water in the soil [29]. Excessive uptake of Na^+^ under high-salinity conditions disrupts the osmotic balance and electrical gradients across cell membranes, leading to disruptions in various physiological processes [3]. Furthermore, the generation of ROS during salinization causes oxidative damage to membranes and cell death [30]. Ultimately, the inhibition of photosynthetic processes [31] and reduction in plant growth and productivity are detrimental consequences of salinization. Nevertheless, genetic engineering’s ability to transfer genes across distinct genetic reservoirs and alter a plant’s native gene expression levels is a potent method for investigating the functionality of genes and creating plants that are more resilient to stress [32]. In a previous study, a yeast screening method was used to identify 69 potential genes associated with drought stress in potatoes [33]. The transformants’ relative tolerance to various abiotic stimuli demonstrated the efficacy of the potato *StD26* gene in conferring drought tolerance and improving the survival rates of yeast transformants [33] Additionally, conserved motifs in the *ARP1* protein were found by several sequence alignments and protein modeling, suggesting significant sequence similarity with proteins from different monocot and dicot species (Figs 1 and 2A). The presence of C2-C2 motifs in *ARP1* suggests its potential involvement in plant responses to abiotic stresses [34], aligning with the known roles of plant C2-C2 domain-containing proteins in various biological processes, as noted by Corbalán-Garcia et al. [35]. Thus, using transgenic *Arabidopsis thaliana*, we were able to clone and functionally characterize the *ARP1* gene to investigate its role in salt tolerance. We examined how transgenic *ARP1* lines responded to 200 mM NaCl in terms of growth, biochemistry, and physiological effects compared to control plants. The observation of healthier, greener leaves in ARP1-L1-NaCl plants compared to the pale-greenish leaves of WT-WW plants indicated the superior growth performance of the transgenic ARP1-L1-NaCl plants under salinity stress (Fig 3D). Additionally, ARP1-L1-NaCl plants exhibited longer roots than ARP1-L1-WW plants (Fig 4D), suggesting that ectopic expression of *ARP1* enables transgenic *Arabidopsis* to better cope with salinity stress in terms of plant growth. Similarly, transgenic tobacco plants expressing *Salicornia brachiate* ribosomal protein displayed greater root and shoot lengths than wild-type plants under salinity stress. The improved performance of the transgenic ARP1-L1-NaCl plants can be explained by their ability to sustain photosynthetic pigment levels and improve photosynthesis in response to salt stress. Indeed, ARP1-L1-NaCl plants exhibited higher levels of chlorophyll a, chlorophyll b, total chlorophyll, and stomatal conductance than ARP1-L1-WW plants under salt stress (Figs 4E and 6). According to Udawat et al. [36], growth related improvement was linked to higher amounts of carotenoids, total chlorophyll, and chlorophyll a in transgenic *ARP1* lines, as well as improved photosynthetic capacity.

Reflectance indices have developed as an efficient method of remotely sensing changes in plant stress levels [37]. Several reflectance indices are vulnerable to drought [38], temperature fluctuations [39], and salinization [40], because their values depend on parameters such as green biomass volume [41] and concentrations of photosynthetic pigments [42]. Photosynthetic activity [43], LAI [44], and various other plant traits that are susceptible to stressors. In particular, the PRI has emerged as a pivotal tool in plant sensing because of its sensitivity to alterations in photosynthetic activity [45] and quick adjustments. Notably, a typical PRI is highly responsive to soil and water salinization [46]. However, directional changes in a typical PRI can depend on the type and severity of stresses [45], thereby constraining its applicability in plant remote sensing. Earlier, researchers have introduced a set of modified reflectance indices based on diverse measurement wavelengths, demonstrating their sensitivity to excessive light, water deficit, and heat. In the current investigation, we discovered that a significant association existed between the maximal quantum yield of photosystem II (Table 1) and most of these indices (NDVI, PSRI, CRI1, WBI, NPCI, ARI1, FRI1, PRI, CCI, and greenness), as well as sensitivity to salinization (200 mM) (Fig 5A–5J). A notable disparity in leaf spectral indices between the WT and *ARP1* lines was evident after salt-induced stress (Fig 5A–5J). However, salinity reduced these indices in WT-NaCl and transgenic ARP1-L1-NaCl and ARP1-L2-NaCl plants compared to their values in WT-WW, ARP1-L1-WW, and ARP1-L2-WW plants. Furthermore, the NDVI, a well-established reflectance index linked to biomass [41, 44], LAI [44], Chl content [47], and other slowly evolving plant parameters, underscored the influence of chlorophyll concentration on the modified PRI. This impact could be caused by changes in the carotenoid-to-chlorophyll concentration ratio, which influences the usual PRI [48].

In current investigation, WT-NaCl plants exhibited unfavorable changes in the structural stability of the PSII center, resulting in a drop in PSII maximal quantum yield (Fv/Fm) and performance index (PI_ABS_) as compared to ARP1-NaCl plants. Plant species typically have an optimal Fv/Fm ratio between 0.79 and 0.83, with lower values indicating stress situations [49, 50]. Therefore, a potential marker for identifying salt-tolerant genotypes is Chl a fluorescence kinetics [51]. An in-depth analysis of the JIP test parameters revealed that, under salinity stress, WT-NaCl plants experienced more significant photoinhibition of PSII than ARP1-L1-NaCl plants, disrupting electron transfer within PSII. Salt stress was less likely to harm transgenic *Arabidopsis* plants expressing potato *StD200* than wild-type plants, which exhibited reduced PSII center stability and a considerable drop in the Fv/Fm ratio [50]. Recent research has shown that salinity inhibits the transfer of electrons from Q_A_ to the electron transport chain, causing the plastoquinone pool (PQH_2_) to decrease, and light dissipation (DIo/RC) to increase sharply [52]. A higher content was needed on the RCs that were still active in our investigation because salt treatment deactivated some of the RCs. The energy dissipation efficiency of the remaining active reaction centers increased as a result of the rising ABS/RC, TRo/RC, and DIo/RC ratios and decreasing ETo/RC values (Table 1). These results were consistent with the effects of salinity observed in *Hordeum vulgare* [49], *Triticum aestivum* [52], and *Raphanus sativus* [53]. Increased DIo/RC levels are also connected with membrane damage, MDA, and ROS generation under stressful conditions [52]. In summary, the previously described findings lend support to the theory that ectopic *ARP1* expression improves PSII activity and photosynthetic pigment levels in transgenic ARP1-NaCl plants, both of which are required for photosynthesis, plant development, and survival in saline environments.

In our investigation, transgenic ARP1-L1-NaCl plants displayed higher proline content than ARP1-L1-WW plants during salinity stress, showing that ARP1-L1-NaCl plants maintain their cytosolic osmotic potential (Fig 10B). Our findings were confirmed by the discovery that transgenic Arabidopsis plants expressing *Withania somnifera* sterol glycosyltransferases 3.1 (*WsSGLT3*.*1*) demonstrated improved salt tolerance and increased proline content than WT plants [54]. Amino acid storage, such as proline accumulation, improves a plant’s resistance to oxidative stress, stabilizes proteins under stress, and preserves cellular turgor [55]. Additionally, the ARP-NaCl plants exhibited higher MDA levels and lower EL percentages than the WT-NaCl plants, suggesting reduced oxidative damage (Figs 4F and 10A). Similarly, *Arabidopsis* plants genetically modified to express *Tamarix hispida* salt overly sensitive 3 (*ThSOS3*) exhibited reduced levels of MDA and EL when subjected to high-salinity conditions compared to their WT counterparts [56]. ROS causes lipid peroxidation within cellular membranes, which produces MDA as a byproduct and acts as a measure of oxidative stress and plant antioxidant capability [57]. Our findings suggest that transgenic *ARP1* lines maintain the integrity of their cell membranes under salinity stress by mitigating the oxidative damage induced by salinity, potentially through enhanced antioxidant mechanisms. Furthermore, ARP1-L1-NaCl plants exhibited greater enzyme activity and expression levels of critical genes involved in antioxidant defense, including APX, CAT, and SOD, in comparison to ARP1-L1-WW plants (Figs 10C–10E and 11). These findings suggest that the ectopic expression of *ARP1* enhances the antioxidant capacity of transgenic plants and helps them maintain their redox/energetic balance in response to salinity stress. This is consistent with other research that demonstrates ribosomal proteins scavenge ROS directly through non-enzymatic antioxidants or indirectly through stimulating the production of genes that encode antioxidant enzymes [36]. Superoxide radicals (O_2_^−·^) are converted by SOD into hydrogen peroxide (H_2_O_2_), which is then neutralized into water (H_2_O) by APX and CAT enzymes [3].

## 5. Conclusion

The results of the present study showed that transgenic *Arabidopsis ARP1* plants with ectopic *ARP1* expression had better salt tolerance, as evidenced by their longer roots than those of WT plants. Our study also revealed a clear correlation between the enhanced growth performance of transgenic *ARP1* plants and *StARP1*-induced improvements in photosynthetic pigments, PSII center maintenance, osmotic adjustment, and cell membrane integrity under salt stress. The higher salt tolerance seen in transgenic *ARP1* lines in comparison to WT plants was partly attributed to the upregulation of stress-responsive and antioxidant enzyme-encoding genes by ectopic expression of *StARP1*. The impact of ectopic *StARP1* expression in *A*. *thaliana* on the ability of plants to overcome salt injuries was examined in the present study. Based on our findings, *StARP1* may be a useful target for the genetic engineering of crops that can withstand saline stress.

## Supporting information

S1 Fig Melting curves plot of Arabidopsis (S1A) Ascorbate peroxidase, (S1B) Catalase, (S1C) superoxide dismutase, (S1D) glyceraldehyde-3-phosphate dehydrogenase and (S1E) potato Auxin repressed protein gene primers. (DOCX)

S1 Table List of the primers used in the study. (DOCX)

S2 Table Formulae and glossary of terms used by the JIP-test for the analysis of Chl *a* fluorescence transient OJIP emitted by dark-adapted photosynthetic samples. (DOCX)

S1 Raw images **S1.** Image of a 1.2% agarose gel showing bands of *Hygromycin phosphotransferase gene* (412 bp) amplified (by PCR) from genomic DNA of different hygromycin-resistant transgenics plants (L1, L2, L3). L1 and L2 corresponds to ARP1-L1 and ARP1-L2 transgenic plants, L3 and L4 corresponds to positive and negative control plants and L5 is DNA ladder. **S2.** Gel picture showing PCR amplified bands corresponding to 136 bp long fragment of *St-ARP1* gene. L1-L11: Lanes, L1: negative control (DNA of wild type *Arabidpsis thaliana*). L2-L5 and L7-L11: test samples (DNA of T1 plants), L2, L4, L8 and L9 are PCR positive, for the tested *St-ARP1* gene. T3 plants of L2 and L3 samples were used as experimental material (based on high StARP1 expression) for further experiments.
(ZIP)
