# Supplementary material for: Ectopic expression of potato ARP1 encoding auxin-repressed protein confers salinity stress tolerance in Arabidopsis thaliana
Source: PLoS One. 2024 Oct 17;19(10):e0309452. doi: 10.1371/journal.pone.0309452 (PMC11486362; doi:10.1371/journal.pone.0309452)
Supplement: S1 Fig — (DOCX) [file pone.0309452.s001.docx]

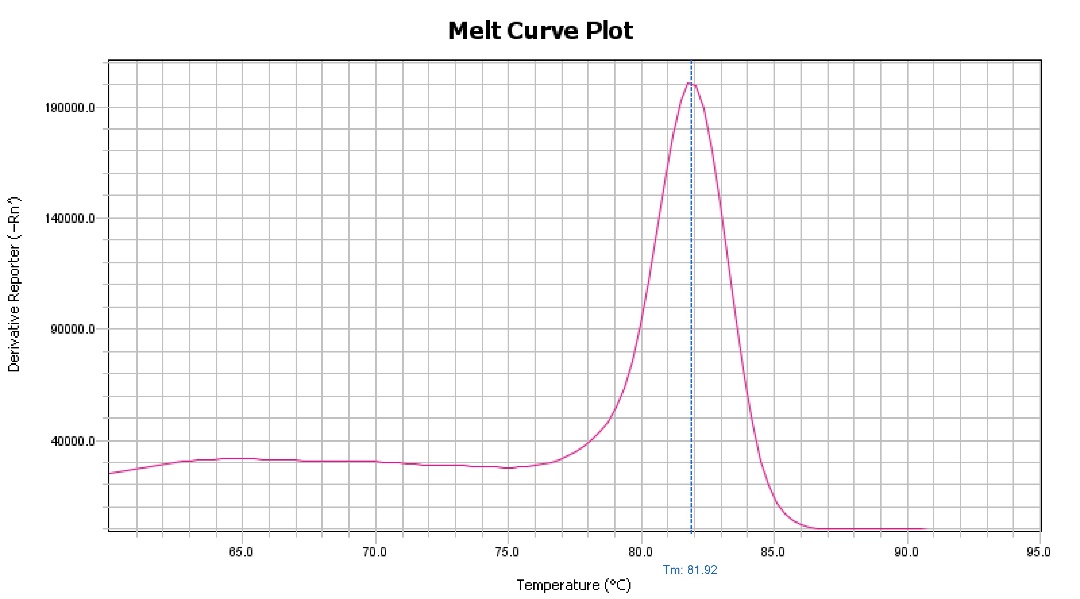


Figure S1A. Melt curve plot of APX gene primers


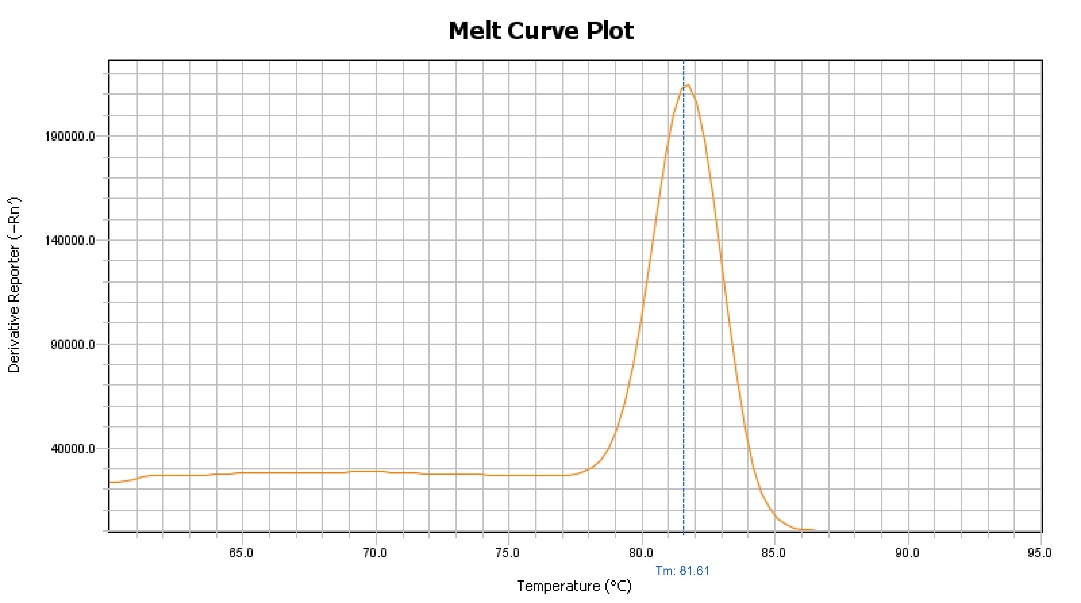


Figure S1B. Melt curve plot of CAT gene primers


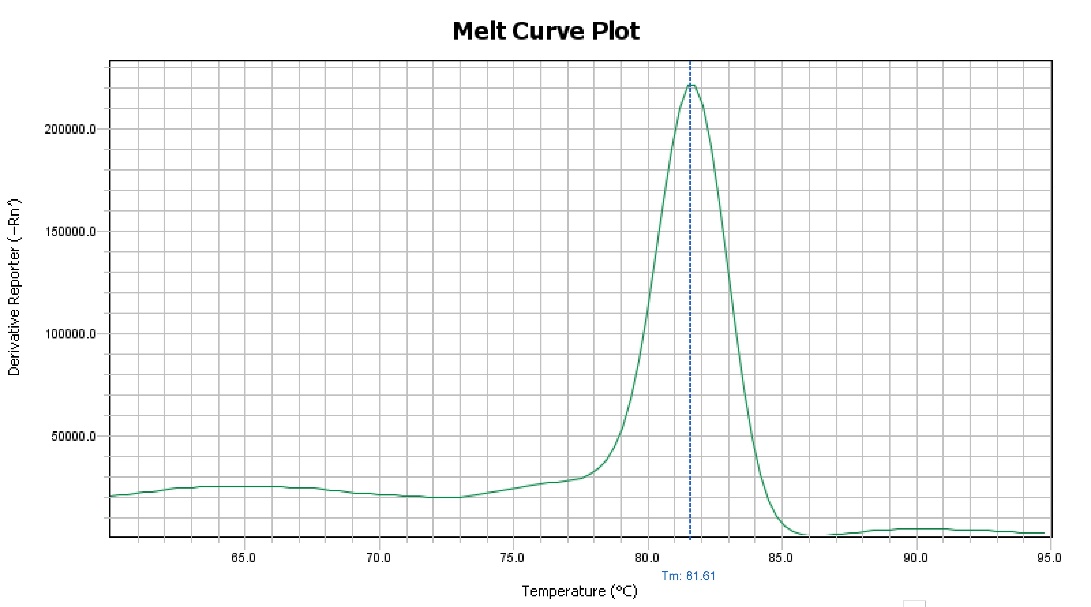


Figure S1C. Melt curve plot of SOD gene primers


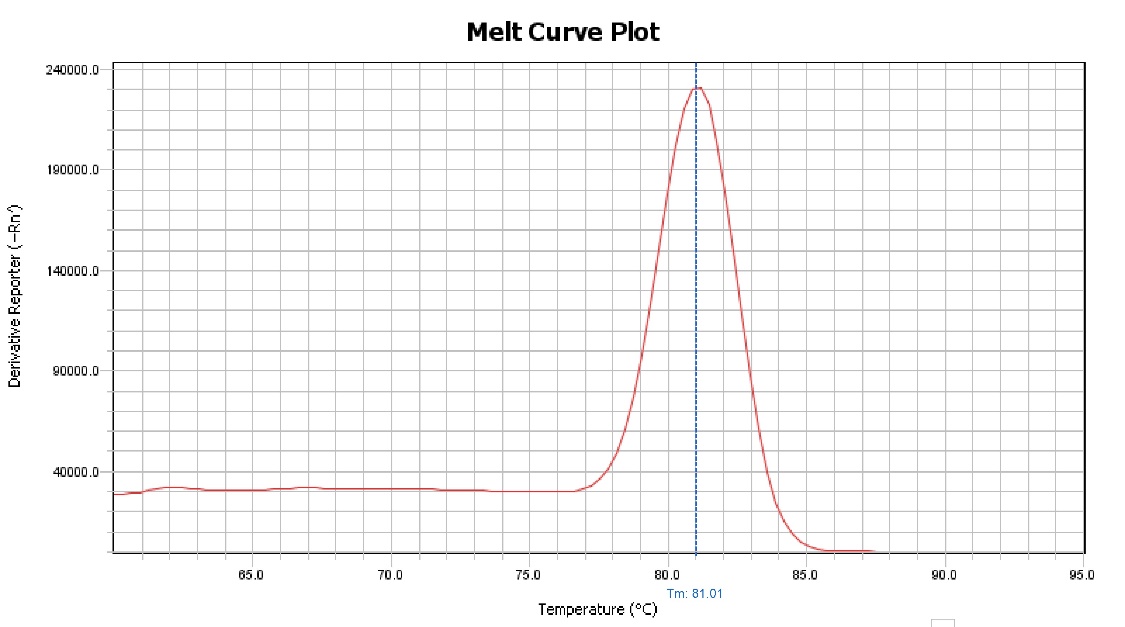


Figure S1D. Melt curve plot of GAPDH gene primers


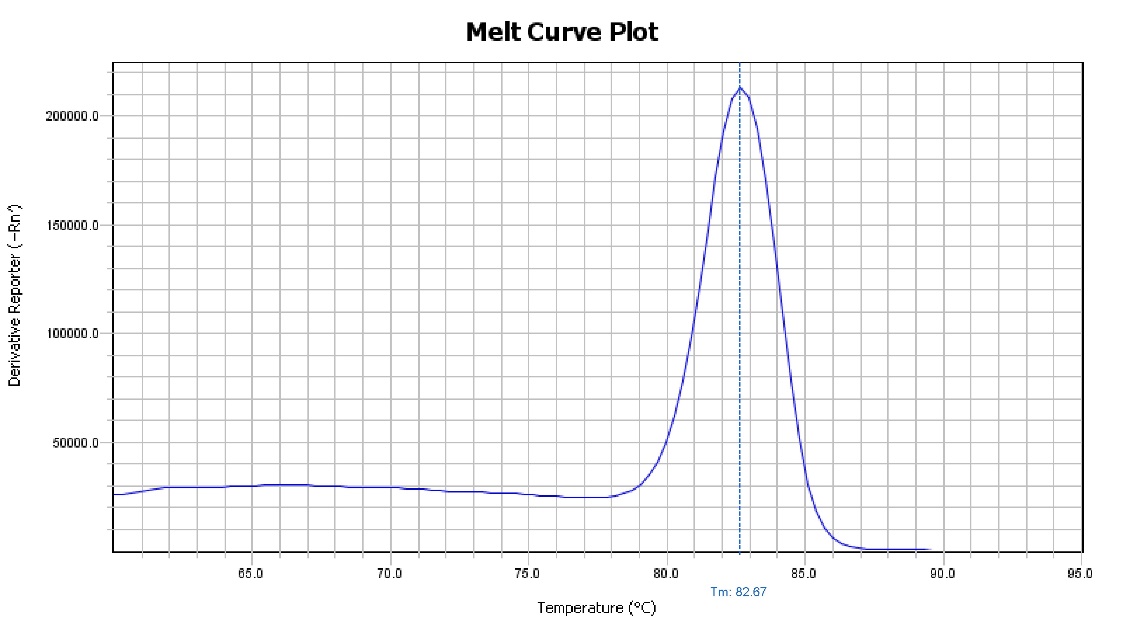


Figure S1E. Melt curve plot of ARP gene primers

**Supplementary Figure S1:** Melting curves plot of Arabidopsis (S3A) Ascorbate peroxidase, (S3B) Catalase, (S3C) superoxide dismutase, (S3D) glyceraldehyde-3-phosphate dehydrogenase and (S3E) potato Auxin repressed protein gene primers.
