# Supplementary material for: Ectopic expression of potato ARP1 encoding auxin-repressed protein confers salinity stress tolerance in Arabidopsis thaliana
Source: PLoS One. 2024 Oct 17;19(10):e0309452. doi: 10.1371/journal.pone.0309452 (PMC11486362; doi:10.1371/journal.pone.0309452)
Supplement: S1 Table — (DOCX) [file pone.0309452.s002.docx]

**Supplementary Table S1**: List of the primers used in the study.

| **Gene name** | **Gene ID** | **Primer** | **Primer Sequence (5’ – 3’)** |
| --- | --- | --- | --- |
| *ARP1** | JX576266 | StARP1_F | CACCATGGTGTTAATTGAA |
|  |  | StARP1_R | TCACTGATGCTTGGATCGGGTAT |
| *Hygromycin phosphotransferase*** | - | PMDCHyg_F | CAGAAGAAGATGTTGGCGACCTCGTA |
|  |  | PMDCHyg_R | TCACGTTGCAAGACCTGCCTGAAAC |
| *ARP1**** | JX576266 | ARP1_F | TCTCTGTCTATGCCGGGCT |
|  |  | ARP1_R | TGGCAATGTTGCTCCCCGGA |
| *Glyceraldehyde-3-phosphate dehydrogenase C2***** | AT1G13440 | GAPDH_F | GAGAGTTTGTGTGTGGTTGAGTTC |
|  |  | GAPDH_R | GGTTTGAGTTAGCACGAGAAAGTAA |
| *Catalase***** | AT1G20630 | CAT_F | AAGTGCTTCATCGGGAAGGA |
|  |  | CAT_R | CTTCAACAAAACGCTTCACGA |
| *Superoxide dismutase***** | AT1G08830 | SOD_F | TCCATGCAGACCCTGATGAC |
|  |  | SOD_R | CCTGGAGACAATGATGCC |
| *Ascorbate peroxidase***** | AT1G07890 | APX_F | CCGTCCTTTGGTCGAGAAATA |
|  |  | APX_R | GGATAAGTACCCAAGCTCAGAAA |

*Used for gene isolation and cloning into the pMDC32 vector

**Used for validation of transgenic plants

*** Used for validation of transgenic plants as well as RT-qPCR

****Used only for RT-qPCR
