# Supplementary material for: Ectopic expression of potato ARP1 encoding auxin-repressed protein confers salinity stress tolerance in Arabidopsis thaliana
Source: PLoS One. 2024 Oct 17;19(10):e0309452. doi: 10.1371/journal.pone.0309452 (PMC11486362; doi:10.1371/journal.pone.0309452)
Supplement: S2 Table — (DOCX) [file pone.0309452.s003.docx]

**Supplementary Table S2**. Formulae and glossary of terms used by the JIP-test for the analysis of Chl *a* fluorescence transient OJIP emitted by dark-adapted photosynthetic samples.

| *Data extracted from the recorded fluorescence transient OJIP* | |
| --- | --- |
| F_t_ | fluorescence at time t after onset of actinic illumination |
| F_50μs_ or F_20μs_ | minimal reliable recorded fluorescence, at 50 μs with the PEA- or 20 μs with the Handy-PEA-fluorimeter |
| F_300μs_ | fluorescence intensity at 300μs |
| F_J_ ≡ F_2ms_ | fluorescence intensity at the J-step (2 ms) of OJIP |
| F_I_ ≡ F_30ms_ | fluorescence intensity at the I-step (30 ms) of OJIP |
| F_P_ | maximal recorded fluorescence intensity, at the peak P of OJIP |
| t_FM_ | time (in ms) to reach the maximal fluorescence intensity F_M_ |
| Area | total complementary area between the fluorescence induction curve and F = F_M_ |
| *Fluorescence parameters derived from the extracted data* | |
| F_0_ ≅ F_50μs_ or ≅ F_20μs_ | minimal fluorescence (all PSII RCs are assumed to be open) |
| F_M_ (= F_P_) | maximal fluorescence, when all PSII RCs are closed (equal to F_P_ when the actinic light intensity is above 500 μmol photons m^-2^ s^-1^ and provided that all RCs are active as Q_A_ reducing) |
| F_υ_ ≡ F_t_ - F_0_ | variable fluorescence at time t |
| F_V_ ≡ F_M_ - F_0_ | maximal variable fluorescence |
| V_t_ ≡ F_υ_/F_V_ ≡ ( F_t_ - F_0_)/(F_M_ - F_0_) | relative variable fluorescence at time t |
| M_0_ ≡ [(ΔF/Δt)_0_]/(F_M_ - F_50μs_)  ≡ 4( F_300μs_-F_50μs_)/(F_M_- F_50μs_) | approximated initial slope (in ms^-1^) of the fluorescence transient normalised on the maximal variable fluorescence F_V_ |
| *Specific energy fluxes (per Q_A_-reducing PSII reaction center - RC)* | |
| ABS /RC = M_0_ (1/V_J_)(1/ϕ_Po_) | absorption flux (of antenna Chls) per RC |
| TR_0_/RC = M_0_ (1/V_J_) | trapped energy flux (leading to Q_A_ reduction) per RC |
| ET_0_/ RC = M_0_ (1/V_J_)ψ_Eo_ | electron transport flux (further than Q_A_^−^) per RC |
| RE_0_/RC = M_0_ (1/V_J_)ψ_Eo_ δ_Ro_ | electron flux reducing end electron acceptors at the PSI acceptor side, per RC |
| *Quantum yields and efficiencies* | |
| ϕ_Pt_ ≡ TR_t_/ABS = [1-(F_t_/F_M_)] = ΔF_t_/F_M_ | quantum yield for primary photochemistry at any time t, according to the general equation of Paillotin (1976) |
| ϕ_Po_ ≡ TR_0_/ABS = [1-(F_0_/F_M_)] | maximum quantum yield for primary photochemistry |
| ψ_Eo_ ≡ ET_0_/TR_0_ = (1-V_J_) | efficiency/probability for electron transport (ET), i.e. efficiency/probability that an electron moves further than Q_A_^−^ |
| ϕ_Eo_ ≡ ET_0_/ABS = [1-(F_0_/F_M_)]ψ_Eo_ | quantum yield for electron transport (ET) |
| δ_Ro_ ≡ RE_0_/ET_0_ = (1-V_I_)/(1-V_J_) | efficiency/probability with which an electron from the intersystem electron carriers moves to reduce end electron acceptors at the PSI acceptor side (RE) |
| ϕ_Ro_ ≡ RE_0_/ABS = [1-(F_0_/F_M_)]ψ_Eo_ δ_Ro_ | quantum yield for reduction of end electron acceptors at the PSI acceptor side (RE) |
| γ_RC_ = Chl_RC_/Chl_total_ = RC/(ABS+RC) | probability that a PSII Chl molecule functions as RC |
| RC/ABS = γ_RC_/(1-γ_RC_ ) = ϕ_Po_ (V_J_/ M_0_) | Q_A_-reducing RCs per PSII antenna Chl (reciprocal of ABS/RC) |
| *Performance indexes (products of terms expressing partial potentials at steps of energy bifurcations)* | |
| PI_ABS_ ≡ __ | performance index (potential) for energy conservation from exciton to the reduction of intersystem electron acceptors |
| PI_total_ ≡ __ | performance index (potential) for energy conservation from exciton to the reduction of PSI end acceptors |
| *Subscript “0” indicates that the parameter refers to the onset of illumination* | |
