# Supplementary material for: Ectopic expression of potato ARP1 encoding auxin-repressed protein confers salinity stress tolerance in Arabidopsis thaliana
Source: PLoS One. 2024 Oct 17;19(10):e0309452. doi: 10.1371/journal.pone.0309452 (PMC11486362; doi:10.1371/journal.pone.0309452)
Supplement: S1 Raw images — S1. Image of a 1.2% agarose gel showing bands of Hygromycin phosphotransferase gene (412 bp) amplified (by PCR) from genomic DNA of different hygromycin-resistant transgenics plants (L1, L2, L3). L1 and L2 corresponds to ARP1-L1 and ARP1-L2 transgenic plants, L3 and L4 corresponds to positive and negative control plants and L5 is DNA ladder. S2. Gel picture showing PCR amplified bands corresponding to 136 bp long fragment of St-ARP1 gene. L1-L11: Lanes, L1: negative control (DNA of wild type Arabidpsis thaliana). L2-L5 and L7-L11: test samples (DNA of T1 plants), L2, L4, L8 and L9 are PCR positive, for the tested St-ARP1 gene. T3 plants of L2 and L3 samples were used as experimental material (based on high StARP1 expression) for further experiments. (ZIP) [file pone.0309452.s004.zip › S1_raw_images/S1_raw_image2.docx]

L1 L2 L3 L4 L5 L6 L7 L8 L9 L10 L11


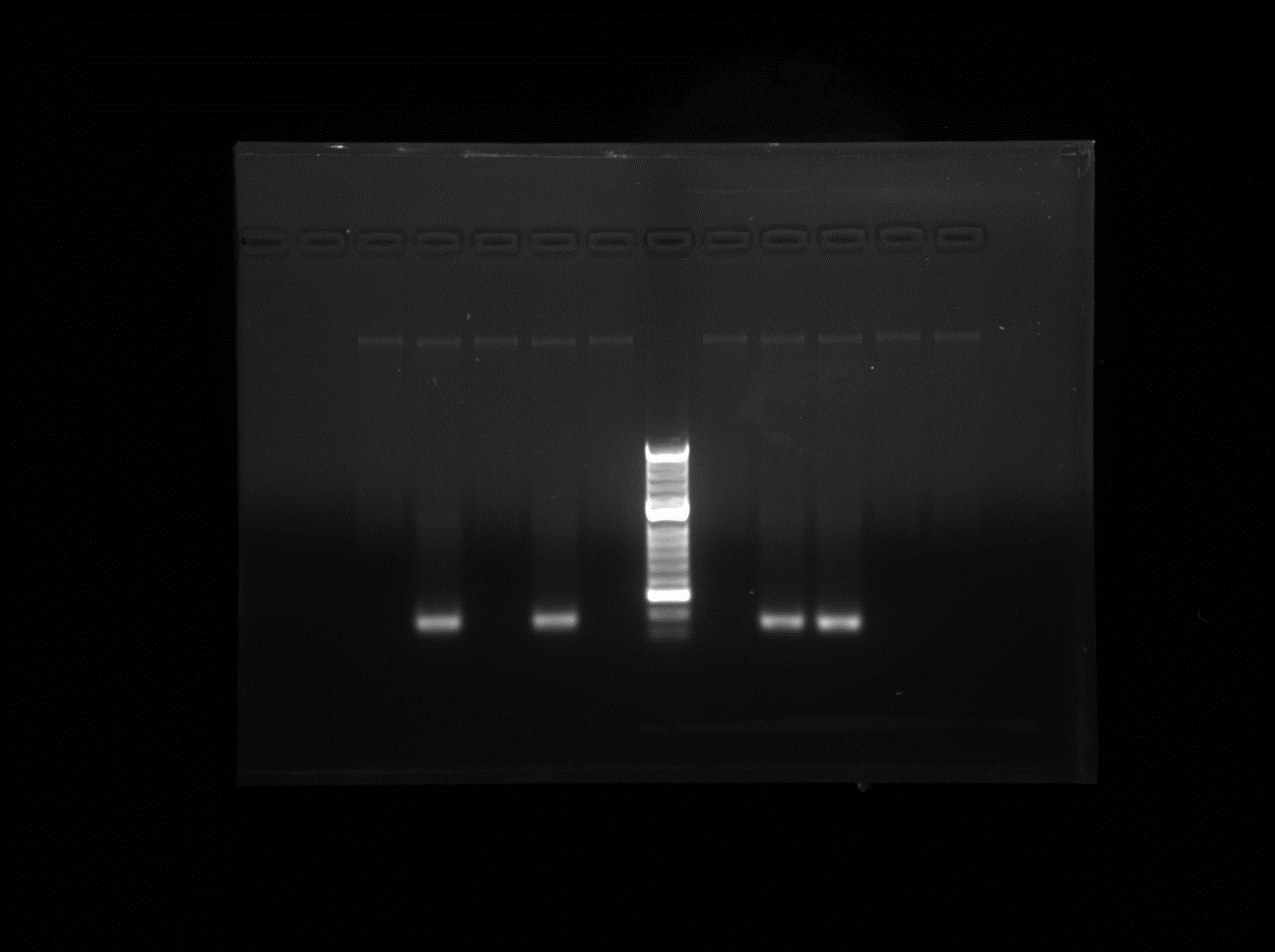


**S1_raw_image S2:** Gel picture showing PCR amplified bands corresponding to 136 bp long fragment of *St-ARP1* gene. L1-L11: Lanes, L1: negative control (DNA of wild type *Arabidpsis thaliana*). L2-L5 and L7-L11: test samples (DNA of T1 plants), L2, L4, L8 and L9 are PCR positive, for the tested *St-ARP1* gene. T3 plants of L2 and L3 samples were used as experimental material (based on high StARP1 expression) for further experiments.
